# Sorption of phosphate onto mesoporous *γ*-alumina studied with in-situ ATR-FTIR spectroscopy

**DOI:** 10.1186/1752-153X-6-26

**Published:** 2012-04-03

**Authors:** Ting-Ting Zheng, Zhong-Xi Sun, Xiao-Fang Yang, Allan Holmgren

**Affiliations:** 1 School of Chemistry and Chemical Engineering, University of Jinan, 250022, Jinan, China; 2 Division of Chemical Engineering, Luleå University of Technology, S-971 87, Luleå, Sweden; 3 Research Center for Eco-Environmental Sciences, Chinese Academy of Sciences, Beijing, China

## Abstract

**Background:**

Due to the extensive use of phosphates in industry, agriculture and households, the phosphate - γ-alumina interactions are important for understanding its detrimental contribution to eutrophication in lakes and rivers. In situ Fourier transform infrared (FTIR) spectroscopy can provide more detailed information on the adsorbate-adsorbent interaction and the formation of hydrogen bonds.

**Results:**

In situ ATR-FTIR spectroscopy was used to identify phosphate complexes adsorbed within the three-dimensional network of mesoporous γ-alumina at pH 4.1 and 9.0. The integrated intensity between 850 cm-1 and 1250 cm-1 was used as a relative measure of the amount of adsorbed phosphate. The integrated intensity proved to be about 3 times higher at pH 4.1 as compared with the corresponding intensity at pH 9.0. The adsorption of phosphate at the two pH conditions could be well described by the Langmuir adsorption isotherm at low concentrations and the empirical Freundlich adsorption isotherm for the whole concentration range, viz. 5 – 2000 μM.

**Conclusions:**

From the band shape of infrared spectra at pH 4.1 and pH 9.0, it was proposed that the symmetry of the inner-sphere surface complex formed between phosphate and γ-alumina was C1 at the lower pH value, whilst the higher value (9.0) implied a surface complex with C2v or C1 symmetry. The difference in adsorbed amount of phosphate at the two pH values was ascribed to the reduced fraction of ≡ AlOH2+ surface sites and the increased fraction of ≡ AlO- sites upon increasing pH from 4 to 9.

## Background

The concern about phosphate distribution in fresh water and soil is motivated by its detrimental contribution to eutrophication in lakes and rivers and the extensive use of phosphates in industry, agriculture and households [1,2]. The mobility of phosphate in soil and aquatic systems is largely determined by the reaction between phosphate and mineral surfaces [3,4]. Because of the abundance of aluminum and iron oxides in soil and sediment and their sorption reactivity to phosphate, the interaction of water with alumina and iron oxide [5-10] and the uptake of phosphate on various Al- and Fe-oxides (e.g., boehmite, gibbsite, goethite, ferrihydrite, hematite) has been studied extensively in the past decades by both macroscopic and spectroscopic methods [11-30]. These investigations showed that phosphate inner-sphere complexes were formed at the metal oxide-water interface within a certain pH range. The type of surface complexes that may form, i.e. monodentate, bidentate mononuclear or binuclear and the degree of protonation varied with pH. The surface coverage was both pH and contact time dependent and in addition to surface complexes also aluminum phosphate precipitates may form.

In situ or ex situ Fourier transform infrared (FTIR) spectroscopy can provide molecular scale information on phosphate configuration at the surface. The type of phosphate complexes that are formed in a fixed aqueous environment may be proposed based on the symmetry of the phosphate complex in combination with curve fitting analysis of the stretching *ν*_3_ vibration of phosphate. However, the bonding of phosphate to the metal oxide surface is difficult to unambiguously predict in detail due to the interplay between protonated surface species and hydrogen bonding. For instance, the protonated monodentate inner-sphere species (≡AlOPO_2_OH) can not be distinguished from monodentate species with a hydrogen bond to an adjacent surface hydroxyl group (≡AlOPO_2_O-HOAl≡), since both species have equivalent low molecular symmetry. In spite of this limitation, FTIR spectroscopy has become one of the most useful tools in the analysis of phosphate species sorbed on solid surfaces. However, IR studies of phosphate sorption onto aluminum oxides are much less common than the corresponding studies on iron oxides, despite the well-known strong interaction between phosphate and aluminum ions implying a high potential to affect the mobility of phosphate in soil systems containing aluminum oxides. Many of these studies were performed using ex-situ methods [23] where spectroscopic data are collected under more or less dry conditions affecting the formation of hydrogen bonds and possibly the adsorbate-adsorbent interaction.

In this study the in-situ Attenuated Total Reflection (ATR) –FTIR spectroscopy technique was used implying that data were collected simultaneously as the adsorbate (orthophosphate) entered the detection volume of the adsorbent deposited on the ATR crystal. The detection volume consisted of a synthesized aluminum oxide with large surface area. A large surface area is advantageous in adsorption studies using the ATR method since the signal to noise ratio is improved implying a lower detection limit for a certain amount of adsorbent. The *γ*-alumina material used in this study improved the signal to noise due to its large surface area and mesoporous structure. The concentration of the total soluble phosphate was very low, down to 5 μM, which was admitted at least at low pH because of this large surface area. The in situ ATR- FTIR method used is also advantageous compared to batch methods since it admits molecular level information of adsorbed species in real time. These advantages facilitated the study of aqueous phosphate adsorption onto mesoporous alumina at both low and high pH and in the concentration range 5 μM to 2000 μM. The objective of the study was to elucidate the structure of complexes formed upon phosphate adsorption on alumina as functions of pH and phosphate concentration and to be able to suggest a possible sorption mechanism based on collected infrared spectroscopic data.

## Results and Discussions

### Characterization

**Figure 1:**
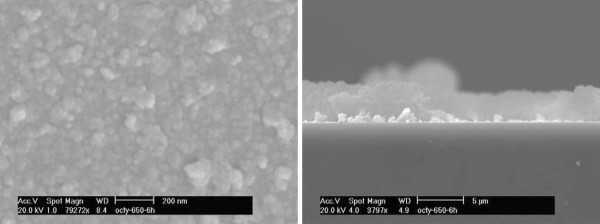
**SEM images of mesoporous alumina deposited on a glass plate (left) top view and (right) side view.** Same amount of adsorbent was deposited on the ATR crystal in the sorption experiments.

Information about structure and surface area of the synthesized mesoporous alumina was obtained from X-Ray Diffraction (XRD) analysis and N_2_(g) adsorption/desorption measurements, respectively. The results showed that the synthesized alumina material had a high surface area *viz.* 329 m^2^⋅g^-1^. The pore size was centered at 9.5 nm with a narrow distribution as shown in Additional file 1: Figure S1 (supporting information) and the pore volume was 0.88 cm^3^ g^-1^. XRD analysis of the synthesized mesoporous alumina sample is presented in Additional file 1: Figure S2, which confirmed the *γ*-Al_2_O_3_ structure [30] (PDF No. 79-1558). The synthesized alumina was dispersed in 0.01 M NaCl aqueous solution, prepared using Milli-Q water, and deposited on a ZnSe ATR crystal for FTIR measurements. The resulting adsorbent layer is shown in Figure 1. The top-view of the Scanning Electron Microscopy image in Figure 1 shows small alumina particles (20-30 nm in diameter) as well as agglomerated particles. The thickness of the deposited alumina layer was around 4 μm, as judged from the side-view image. The distinctly white part is fractures from the substrate.

It is well-known that upon attenuated total reflection at the surface of an ATR crystal, the electric field will decline exponentially with the distance from the crystal surface. Accordingly, phosphate adsorbed in the mesoporous alumina layer closer to the reflecting surface will contribute more to the recorded infrared absorption than phosphate species adsorbed in the outer part of the detection volume. The penetration depth is the distance from the crystal surface where the electric field has dropped to e^-1^ of its value but the electric field is of course larger than zero also beyond that distance. For a ZnSe crystal, the penetration depth is ~1.6 μm at 1000 cm^-1^ provided the refractive index of the porous alumina layer in water is ~1.4. However, a deposited layer thicker than the penetration depth is rather an advantage here since the intensity recorded from adsorbed species will not be much influenced by the layer being 3 μm or 5 μm thick.

### Effect of pH and concentration on phosphate sorption

**Figure 2:**
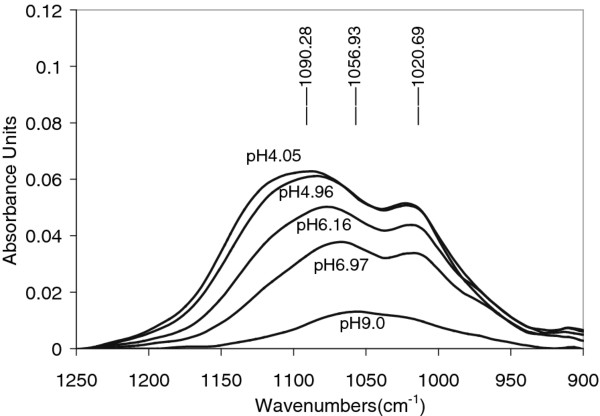
**Infrared spectra showing phosphate adsorbed on mesoporous*γ*-alumina from a 0.050 mM aqueous phosphate solution at pH: 4.05, 4.96, 6.16, 6.97, and 9.0.** The absorption intensity decreased with increasing pH.

Figure 2 shows spectra of phosphate sorbed onto *γ*-alumina from 50 μM aqueous phosphate solution at various pH. The integrated infrared absorption between 1250 and 850 cm^-1^, which corresponds to the stretching frequency region of phosphate species is related to the amount of adsorbed phosphate. The spectral intensity increased from pH of 9.0 to pH of 4.05, indicating that acidic pH favors phosphate adsorption in accordance with previous studies. The spectral line shape changed and the peak position shifted from 1056 cm^-1^ at pH 9.0 to 1090 cm^-1^ at pH 4.05. In addition, a new strong band appeared at 1020 cm^-1^. It is clear from this figure that the amount of phosphate adsorbed increased with decreasing pH.

**Figure 3:**
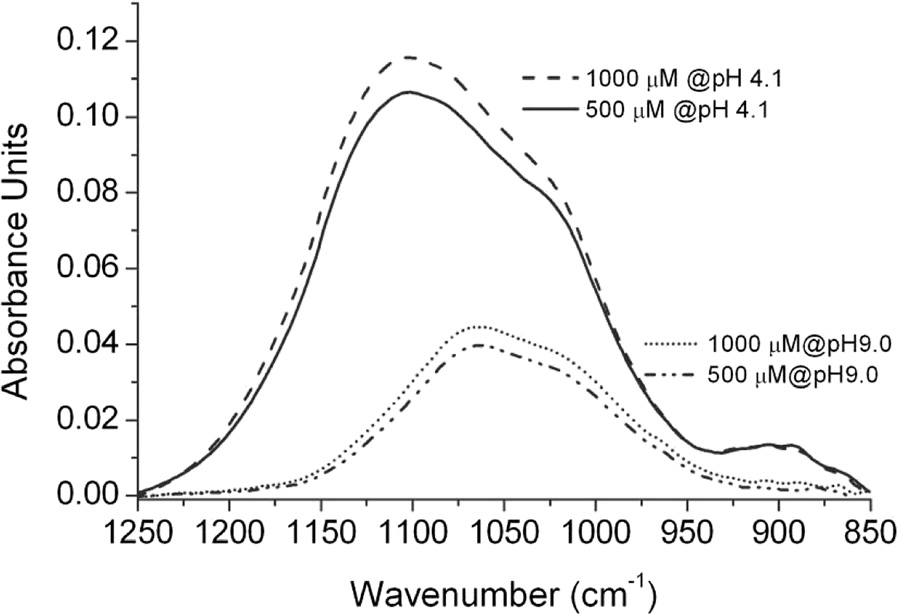
**Infrared spectra of sorbed phosphate recorded from 1.0 mM and 0.5 mM aqueous phosphate solutions onto*γ*-alumina.** The two upper curves are recorded at pH = 4.1 (after 20 min) and the lower curves at pH = 9.0 (after 20 min).

Figure 3 shows infrared spectra of phosphate sorbed onto γ-alumina from 1000 μM and 500 μM aqueous phosphate solutions at pH 4.1 and pH 9.0. According to Figure 3, the band intensity increased with the phosphate concentration in solution. At each of the two pH values, the line shapes of the infrared bands are very similar at the two phosphate concentrations shown although the bands became broader at higher concentration. The band intensity increased much faster with time upon sorption from a phosphate solution at pH 4.1 compared with sorption at pH 9.0. However, the amount of adsorbed phosphate did not reach an equilibrium plateau value but rather a constant slow increase in the adsorbed amount with time (~2 % in spectral intensity at pH 4.1). This increase with time may be due to the formation of aluminum phosphate. The increase with time was smaller at pH 9.0 compared with pH 4.1 and the beginning of the constant adsorption rate region was used for a Langmuir type of data evaluation. The onset of this region was assumed to represent an equilibrium sub-monolayer adsorption of phosphate.

As calculated from the thermodynamic equilibrium constants, H_2_PO_4_^-^ and HPO_4_^2-^ are the dominating species in solution at pH 4.1 and 9.0, respectively (see Additional file 1: Figure S3). Therefore, the relative amounts of adsorbed phosphate at the two pH values could be estimated assuming that equal amounts of *γ*-Al_2_O_3_ was penetrated in each experiment, which also was supported by the pH-envelope experiments where the same alumina layer was exposed to phosphate in solution starting at pH 9.0 and ending at pH 4.1 (see Figure 2). A layer thickness larger than the penetration depth seems to facilitate this estimation. However, it is not straight forward to compare the amount of adsorbed H_2_PO_4_^-^ and the amount of absorbed HPO_4_^2-^ because the transition moments of the two adsorbed species may be different implying different absorption coefficients. To approach this problem, the absorption of the two species in solution was compared. It turned out that the same concentration (10000 μM) of the two species resulted in a slightly higher integrated absorbance for HPO_4_^2-^ as compared with H_2_PO_4_^-^ and that the splitting of the ν_3_ vibration is different because of different symmetries (C_3*v*_ and C_2*v*_). According to the Beer-Lambert Law, it implies that the absorption coefficient for H_2_PO_4_^-^ in solution should be slightly lower than this coefficient for HPO_4_^2-^ in solution. A first assumption would be that the absorption coefficients of these two species, when forming surface complexes, are either close to similar or the coefficient of adsorbed H_2_PO_4_^-^ is rather lower than the corresponding coefficient for adsorbed HPO_4_^2-^. In either case, it seems reasonable to suggest that the amount of phosphate adsorbed at the lower pH value is much larger than the amount adsorbed at the higher pH value as indicated in Figure 3.

This relation between the two adsorbed species at low and high pH is also supported by batch experiments [11]. It is also supported by Johnson et al. in their study of phosphate adsorption onto *γ*-Al_2_O_3_ using NMR spectroscopy [28]. The latter investigation indicates that the amount of phosphate adsorbed at the lower pH should be about a factor of two larger than at the higher pH value, compared to a factor of about 3 estimated from our infrared spectra (Figure 3) assuming that the ratio between the absorption coefficients for the two species in solution is similar to the adsorbed species. It is clear from these comparisons that the transition moments of the phosphate species in solution change upon adsorption.

### Spectral simulation

**Figure 4:**
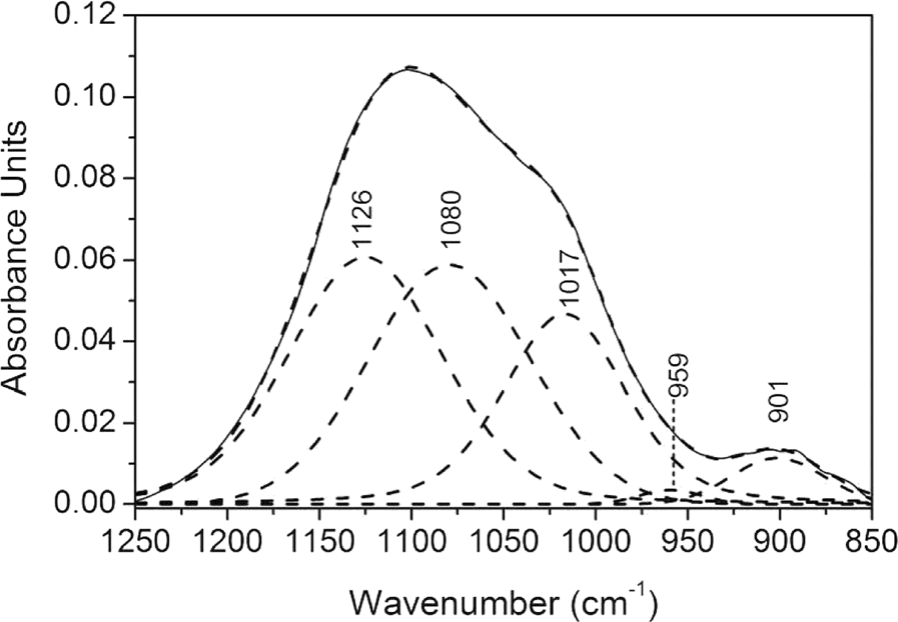
**Infrared spectrum recorded for phosphate adsorbed onto mesoporous γ-alumina from a 500 μM phosphate solution at pH = 4.1 (upper curve as solid line).** The adsorption time was 20 minutes. The five curve fitted sub-bands (dashed lines) shown resulted in a residual root mean square (RMS) error of 0.00069.

The line shape of the infrared absorption representing adsorbed H_2_PO_4_^-^ resulted in five sub-bands, as obtained by the band simulation. This is shown in Figure 4, where the spectral line shape of phosphate adsorbed from 500 μM phosphate solution at pH 4.1 was simulated by curve fitting. The five sub-bands were centered at the frequency positions 1126, 1080, 1017, 959, and 901 cm^-1^. However, five sub-bands is at least one too many since both C_2*v*_ and C_1_ symmetry is expected to result in three bands from the triply degenerate asymmetric stretching and one band from the active symmetric stretching of the phosphate entity. Alternatively, the bands may be caused by simultaneous detection of both inner-sphere and outer-sphere complexes.

Excluding the band at 1080 cm^-1^, this result is fairly consistent with previous studies of phosphate adsorption onto, ferrihydrite [16], hematite [17] and goethite [18] from aqueous solution, which suggested that the dominant surface complex at low pH was (FeO)_2_-PO-OH. The *ν*_3_ peak positions in these studies were obtained at about 1120, 1006, and 970 cm^-1^, where the highest frequency was assigned to a mode involving the P = O double bond. Some difference in frequency positions, compared to alumina, was expected since the adsorption sites are represented by two different metal atoms although the valence of the two is identical. However, according to our findings there is also an absorption band located at ~1080 cm^-1^ that may not to be due to the splitting of the *ν*_3_ vibration. According to calculations using density functional theory (DFT) by Kwon and Kubicki [29] on phosphate adsorbed onto iron hydroxides, the most probable structure was found to be a diprotonated bidentate binuclear complex at low pH (4.2 – 5.7). The calculated vibration frequencies showed for example absorptions at 1120, 1080, 993, and 940 cm^-1^. Comparing our experimental results for the adsorption of phosphate onto mesoporous alumina with results from computed infrared frequencies from phosphate adsorbed on iron oxide [29] and experimental results from phosphate adsorption onto iron oxides [16-18], the diprotonated bidentate binuclear complex was excluded. There are well documented experimental results showing that the frequency appearing at 1126 cm^-1^ in our experimental spectra should be mainly due to the P = O entity. Accordingly, the dominating surface complex at pH 4.1 would be a monoprotonated bridging complex or a diprotonated monodentate complex. Moreover, the calculated frequencies of the monoprotonated bridging phosphate complex with Fe-hydroxide implied an absorption at 1113 cm^-1^, which is close to the expected frequency of the P = O stretch that should show up in this surface complex. The difference from our experimental value (1126 cm^-1^) is about 1 %, which to our experience is very small, especially since the surface site atoms are different (Fe instead of Al). Due to the appearance of the latter frequency in our spectra at low pH, it seems reasonable to suggest that the surface complex formed between mesoporous *γ*-Al_2_O_3_ and phosphate at low pH is a monoprotonated bidentate binuclear (AlO)_2_-PO-OH complex or a diprotonated monodentate complex. The sub-band at 1080 cm^-1^ should at least partly be due to precipitated aluminum phosphate. In a separate experiment the infrared spectrum of AlPO_4_ nH_2_O was recorded. A broad infrared absorption was obtained with maximum absorbance at ~1087 cm^-1^. Subtraction of this band from the total band shape in Figure 4 (pH 4.1), resulted in only minor wavenumber shifts of the simulated bands but in addition a band of low intensity appeared at about 1250 cm^-1^. The latter band may be due to PO-H bending vibration indicating that the complex formed is protonated.

**Figure 5:**
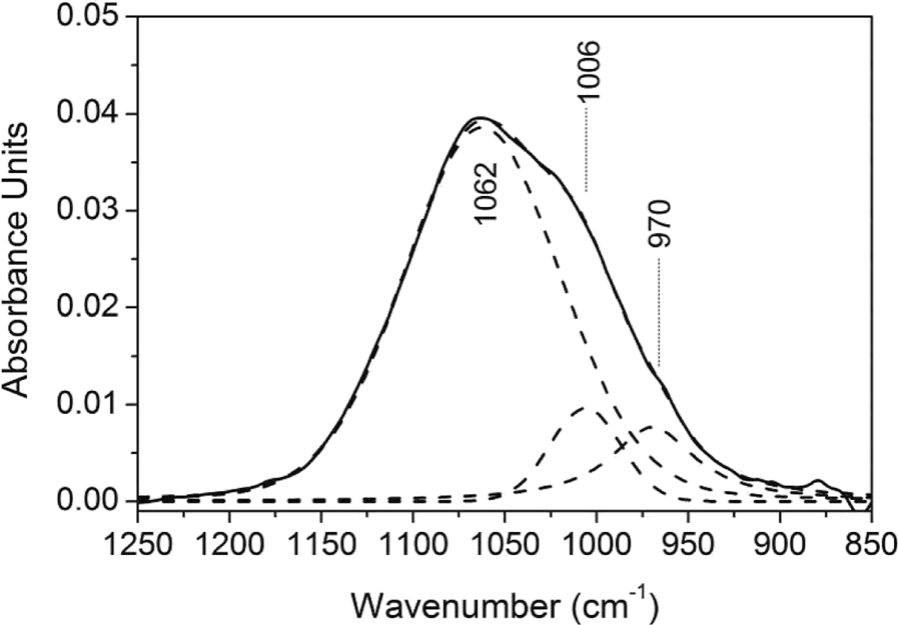
**The infrared band of phosphate adsorbed onto*γ*-Al**_**2**_**O**_**3**_**from an aqueous 0.5 mM phosphate solution at pH 9.0 after 20 minutes of adsorption.** The fitted sub-bands resulted in a RMS error of 0.0004.

Figure 5 shows the simulated bands for phosphate adsorption from a 500 μM phosphate solution at pH 9.0 after 20 minutes of sorption. The curve fitted sub-bands were located at 1062, 1006, and 970 cm^-1^. The number of sub-bands at higher frequency than 900 cm^-1^ was reduced from four to three, without any contribution from precipitated aluminum phosphate at 1087 cm^-1^, which means that from a symmetry point of view the surface complexes would have C_2*v*_ or C_1_ symmetry at pH 9.0. However, at this pH the band at 1120 cm^-1^, corresponding to P = O stretching, is missing and therefore a nonprotonated bidentate binuclear or a monoprotonated monodentate complex seems to be the most reasonable phosphate configurations at the mesoporous *γ*-alumina surface.

It may be added that normalized absorbance spectra (not shown) recorded for phosphate concentrations from 5 μM to 2000 μM (at pH 4.1) showed that; the peak frequency shifted to higher wavenumber (~15 cm^-1^), the infrared bands became slightly broader with increasing phosphate concentration, and that the increased half-width was caused by an increased broadening only on the high wavenumber side of the band. The shift of the peak frequency became evident at high phosphate concentration (see Additional file 1: Figure S5). The reason for this change of the line shape at high phosphate concentration is ascribed to aluminum phosphate precipitation, as discussed below.

### Sorption isotherm

**Figure 6:**
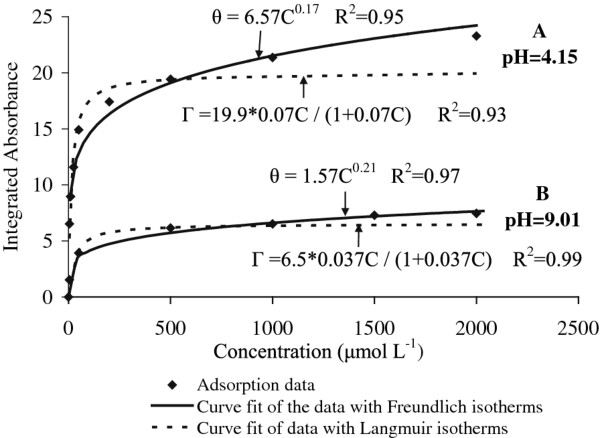
**Adsorption isotherms of phosphate at pH 4.1 (A) and 9.0 (B) upon adsorption at the water-mesoporous*γ*-alumina interface.** The solid and dotted lines are the results of fitting using the Freundlich and the Langmuir models, respectively. IA denotes Integrated Absorbance.

**Figure 7:**
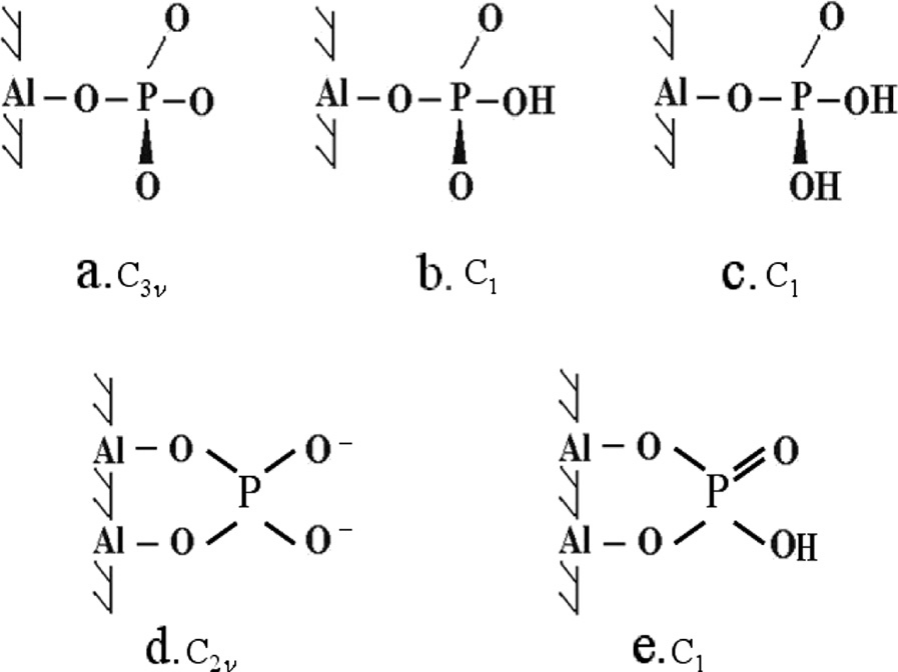
Possible molecular configurations of P inner-sphere complexes on the alumina-water interface at various pH values; a) nonprotonated monodentate, b) monoprotonated monodentate, c) diprotonated monodentate, d) nonprotonated bidentate binuclear, e) monoprotonated bidentate binuclear.

The integrated absorption as a function of concentration at each of the two pH values is plotted in Figure 6. It was evident that the amount of phosphate adsorbed on aluminum oxide increased with increasing phosphate concentration but the increments between equilibrium plateau values became gradually smaller at higher concentrations. The adsorption data were adapted to the Langmuir and Freundlich isotherm models, as shown in Figure 6(A) and (B). Evidently, the adsorption data fits better to the Langmuir isotherm in the low concentration range and the Freundlich isotherm at higher concentrations although the latter isotherm seems to be more suitable to describe the adsorption behavior for the whole concentration range. Guan et al [31] suggested that the Freundlich isotherm model indicates the heterogeneity of the adsorbent. In our study, this adsorption behavior was obtained irrespective of the amount of *γ*-alumina deposited on the ATR crystal. Comparing the integrated absorbencies, the amount of phosphate adsorbed at pH 4.1 was about 3 times as high as the amount adsorbed at pH 9.0. The reduced adsorption of phosphate at pH 9.0 is most likely due to the absence of ≡ AlOH_2_^+^ sites and the formation of ≡ AlO^-^ sites at the higher pH value. The adsorbate is negatively charged at both pH 4.1 and 9.0. Figure 7 shows a few more or less realistic molecular symmetries that are possible for the Al–P complexes.

The adsorption of phosphate at the surface of alumina has been studied by different methods and the proposed types of binding differ from monodentate to bidentate upon varying the solution pH and surface coverage. However, it seems that the monodentate type of phosphate binding to the aluminum and iron oxide surface is often suggested [32]. For example in the adsorption of phenyl phosphonic acid on the alumina surface a strong adsorption was observed at pH < 7, whereas a strong decrease in adsorption affinity was observed within the approximate pH range of 7.7 - 9.5 [33], The results of phosphate adsorption at the surface of *γ*-Al_2_O_3_ in this study showed a tendency similar to that of the adsorption of phenyl phosphonic acid onto alumina, although phosphate here was still adsorbed at the higher pH limit. However, the lack of a band due to P = O stretching at high pH suggests a bidentate bridging or a monodentate type of binding upon increasing the solution pH. The monodentate type of complex might be preferred since a free rotation about the Al–O bond may facilitate hydrogen bonding interaction.

Taking all these pieces of information into account, including the higher density of adsorbed phosphate at low pH, it is suggested that the inner-sphere surface complex between phosphate and the current alumina surface is of the diprotonated monodentate type or a monoprotonated bidentate binuclear complex at pH 4. In addition, precipitated aluminum phosphate seemed to be formed at this pH, which also is in accordance with results from previous NMR measurements [28]. At a pH value of 9, the infrared spectra of the adsorbed complexes are in accordance with a nonprotonated bidentate binuclear or a monoprotonated monodentate configuration.

### Mechanism of sorption

As already pointed out the amount of phosphate adsorbed at pH 4.1 is about 3 times as large as the amount adsorbed at pH 9.0 (Figure 3). This is supported by the simulation of the protonation state of adsorbent surface sites (as shown in Additional file 1: Figure S4), suggesting that the number of ≡ AlOH_2_^+^ sites at the solid surface is reduced substantially upon increasing the solution pH from 4.1 to 9.0 and therefore the observed reduced intensity of adsorbed phosphate at the higher pH value might be explained by the reduced availability of positive adsorption sites along with an increasing number of negatively charged surface sites (see Additional file 1: Figure S4).

However, the ratio between the amount of adsorbed phosphate at pH 4.1 and pH 9.0 (~3) increases with the bulk concentration of phosphate (Figure 6) largely caused by the increased infrared intensity with phosphate concentration, at pH 4.1. This increase in intensity can not be due to infrared absorption from anions in bulk solution considering the thick (~4 μm) alumina layer on the ATR crystal, but could be interpreted as an increased aluminum phosphate precipitation.

It seems also reasonable to assume that phosphate entities in solution take part in hydrogen bonding to already adsorbed inner-sphere complexes thereby forming outer-sphere complexes. Most likely the negatively charged part of the H_2_PO_4_^-^ entity takes part in this interaction with the diprotonated or monoprotonated non-charged surface complexes. The hydrogen bonded H_2_PO_4_^-^ anion has C_2v_ symmetry in aqueous solution since three υ_3_ bands were observed at 1159, 1074, and 939 cm^-1^. The 1074 cm^-1^ band had the strongest absorbance.

The symmetry and the peak positions are expected to be the same when H_2_PO_4_^-^ is hydrogen bonded to an inner-sphere complex. Accordingly, the strong absorption at about 1080 cm^-1^ (Figure 4) may, in addition to precipitated aluminum phosphate, also be caused by contribution from hydrogen bonded phosphate species in outer-sphere position.

At pH 9.0, the surface complex is supposed to be nonprotonated or monoprotonated implying that the negatively charged surface complex offers hydrogen bonding possibilities to the proton of the HPO_4_^2-^ anion. However, this interaction is prohibited by the repulsion between the negatively charged species adsorbed at the alumina surface and the anion dominating in the bulk solution (HPO_4_^2-^) and therefore the increase in bulk phosphate concentration does imply only a small increase in adsorbed amount of phosphate (Figure 6).

**Figure 8:**
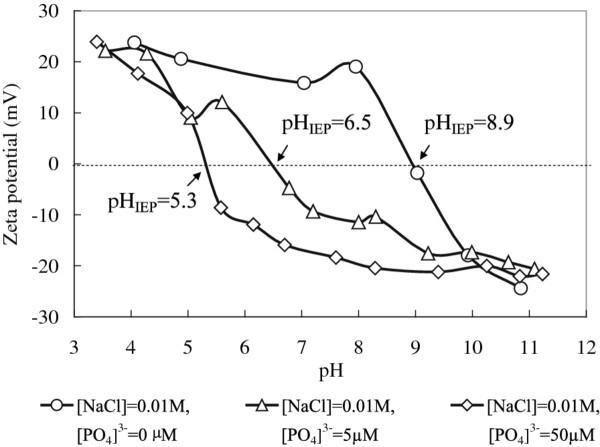
**Zeta potential of mesoporous*γ*-Al**_**2**_**O**_**3**_**in the absence and presence of phosphates.** Phosphate concentrations and pH values are indicated.

A support for this interpretation of the sorption mechanism at pH 9.0 is the results from zeta potential measurements using the electrophoresis method. The results of the zeta potential of the synthesized mesoporous *γ*-Al_2_O_3_ in the absence and presence of phosphates as a function of pH are presented in Figure 8. These measurements showed that the iso-electric point (IEP) of the mesoporous alumina particles dispersed in aqueous 0.01 M NaCl solution was close to pH 9.0. However, in a 5 μM aqueous phosphate solution at pH 9.0, the zeta potential became negative (- 15 mV) and decreased to - 20 mV in a 50 μM solution. This finding became a support for the suggested surface complexes formed at pH 9.0. Furthermore, it offered an explanation to the sorption of negatively charged phosphate species at this pH since the alumina surface was about neutral to start with implying a large number of OH groups at the mesoporous surface. However, during the adsorption of phosphate the surface charge becomes more and more negative and accordingly the continued adsorption of negatively charged phosphate species should be increasingly prohibited at higher bulk concentrations of phosphate.

At pH 4.1, the zeta potential measurements showed the alumina surface to be positively charged irrespective of the bulk concentration of phosphate being 5 μM or 50 μM. The zeta potential measured at 5 μM (~20 mV) only decreased by a few mV upon increasing the phosphate concentration to 50 μM. At this phosphate concentration the number of surface sites on the alumina surface (assuming 1 site per nm^2^) is far below the number of H_2_PO_4_^-^ units in the aqueous solution but the concentration of protons at this pH exceeds the concentration of phosphate anions (50 μM) by a factor of 2. However, it seems reasonable that the number of adsorbed species at pH 4.1 is much larger than at pH 9.0 since a positively charged surface should be more prone to accept negatively charged species.

## Experimental

### Synthesis and preparation of alumina layer for IR analysis

The hydrous mesoporous alumina was prepared by adding NH_3_·H_2_O solution (2.5%) slowly to a rapidly stirred AlCl_3_·6H_2_O solution (1 mol⋅L^−1^). PEG (relative molecular mass = 4000) solution was used as a structure-directing reagent and as a dispersant to prevent the solid particles from aggregation. In addition dodecylamine was used as co-template. The suspension was centrifuged after being intensively stirred for 1 h at pH 9.0. The centrifugate was then rinsed with distilled water and ethanol in order to remove extra PEG and other impurities. The resulting gel was dissolved in ethanol solution and distilled at 100 ^°^C to remove the alcohol. In the last step, mesoporous *γ*-alumina was obtained by calcination of the aluminum hydroxide powder at 650^°^C for 6 hours. The resulting adsorbent had a N_2_ BET surface area of 329 m^2^/g, which may be compared with a common surface area of *γ*-Al_2_O_3_ offered by suppliers, *viz.* 140 m^2^/g.

Stock alumina dispersions (0.5 g⋅L^-1^) were prepared by dispersing solid mesoporous *γ*-alumina in 0.01 M NaCl solution. The resulting dispersion was shaken during one hour. 2.0 ml (≈1 mg) of the alumina dispersion was evenly distributed over one side of the trapezoidal (50 x 20 x 3 mm) ZnSe ATR crystal (45^o^ bevel) by a pipettor and subsequently dried in air overnight to deposit a dry alumina layer. The oxide layer was then rinsed with water to eliminate the particles that were loosely adhered to the crystal.

### In situ ATR-FTIR measurements

The experimental setup for in situ ATR measurements is described in more detail elsewhere [27]. The dimension of the ATR crystal and the experimental setup used allowed 10 reflections to penetrate the mesoporous layer. The refractive index of the ZnSe crystal was 2.41 implying a penetration depth of 1.6 μm at 1000 cm^-1^ assuming a refractive index of 1.4 for the porous *γ*-Al_2_O_3_ phase on the crystal surface. Since the porous alumina layer is assumed to be penetrated by water, the refractive index of the alumina/water phase was estimated from the refractive index of water at 1084 cm^-1^ and the refractive index of γ-alumina (1.6). Data for the phosphate adsorption isotherm were obtained at fixed pH values of 9.0 and 4.1. Phosphate solutions were prepared from Milli-Q water and NaH_2_PO_4_^.^H_2_O (Sigma-Aldrich). For these experiments, the alumina deposit was first equilibrated with 0.01M NaCl as ionic medium adjusted to the pH of interest and subsequently a single beam background spectrum was recorded. Then, the aqueous phosphate solution was allowed to flow over the alumina layer at a rate of 1 mL min^-1^ (0.01 M NaCl as ionic medium). Spectra of phosphate adsorbed onto the alumina layer were recorded every ten minutes. After an initially fast adsorption, a constant slow adsorption rate was obtained, which was confirmed by the difference in the spectral intensities of two successive spectra (less than 2%). A small volume of concentrated phosphate stock solution was added to increase the phosphate concentration stepwise. The initial concentration of phosphate at each step was used for isotherm data evaluation, since the amount of phosphate in solution was in excess relative to the amount of surface sites, due to the small amount of alumina deposited. Assuming a surface site density of 1 site/nm^2^, only for the lowest phosphate concentration (5 μM) the number of phosphate entities in solution corresponds to the number of surface sites in the detection volume. The incremental increase in phosphate concentration or change of pH was accomplished through an external reaction vessel implying that a set of adsorption experiments could be performed on the same layer of *γ*-alumina.

Experiments using a non-coated ZnSe crystal indicated that contributions from aqueous phosphate to the IR spectrum became visible above the noise level only at solution concentrations higher than 1000 μM. However, the amount of *γ*-alumina deposited on the ATR crystal was large enough to prohibit most of the infrared field to reach into the bulk solution and therefore all spectra are dominated by absorptions caused by alumina-sorbed phosphate complexes with a negligible contribution from phosphate in the bulk. Infrared spectra were recorded on a Bruker 66v/S FTIR spectrometer equipped with a DTGS detector and at room temperature (23 ± 1^o^C) and atmospheric pressure using the double side forward-backward acquisition mode. A total number of 128 scans were signal-averaged using an optical resolution of 4 cm^-1^. The resultant interferogram was Fourier transformed using the Mertz phase correction mode, a Blackman-Harris 3-term apodization function, and a zero filling factor of 2. The recorded intensity of adsorbed phosphate at each bulk concentration was determined by integrating the absorption due to phosphate between 1250 cm^-1^ and 850 cm^-1^.

### Zeta potential measurements

The zeta potential measurements were carried out on mesoporous γ-alumina sample using Zeta Compact zeta potential analyzer (CAD, France). An aliquot of the sample suspension with or without phosphate was dispersed in 40 mL NaCl solution. The concentration of γ-alumina in the dispersion was ca. 5 mg·L^-1^. The pH was adjusted with diluted NaOH and HCl solution. The conditioning time corresponds to that of FTIR measurements. After conditioning, the solution pH was measured and the electrophoretic mobility of particles was recorded and further processed by the Zeta 4 software applying the Smoluchowski equation. For each sample, the measurement was repeated three times and the final zeta potential was calculated as an average of the obtained values.

### Speciation modeling

The speciation modeling of phosphate species in solution was carried out using computer program MEDUSA and the relevant equilibrium constants were collected from the MEDUSA database [34]. The speciation of mesoporous γ-alumina surfaces was obtained using computer software WinSGW [35]; the surface acid base equilibrium constant for mesoporous γ-alumina was taken from our own published results [36].

## Conclusions

Adsorption of phosphate onto synthesized mesoporous γ-alumina was characterized at acid (pH 4.1) and basic pH (9.0) by in-situ ATR-FTIR spectroscopy. The results indicated that different phosphate surface complexes were formed at the two pH values. At low pH, infrared spectra could be interpreted as a diprotonated monodentate inner-sphere complex formed together with outer-sphere complexes hydrogen bonded to the already formed inner-sphere complexes. However, a monoprotonaded bidentate binuclear inner-sphere complex with the same symmetry could not be excluded implying that both types of inner-sphere complexes can appear simultaneously on the γ-alumina surface. In addition, precipitated AlPO_4_ nH_2_O was formed on the γ-alumina surface at this low pH and started to form already in the beginning of the adsorption reaction as judged from the smooth change of the band shape with increasing phosphate concentration.

The amount of adsorbed phosphate increased rapidly at high phosphate concentrations and short reaction times followed by slow and small constant increase at longer reaction times. At pH 4.1 an equilibrium plateau value was not reached even at long reaction times (> 300 min) probably due to precipitation of aluminum phosphate.

At pH 9.0, the amount of adsorbed phosphate was only about 33 % of the amount adsorbed at pH 4.1, provided the transition moment of the surface complexes formed at the two pH values are about similar. This difference in adsorbed amount increased slightly at higher bulk phosphate concentrations (> 500 μM).

At the highest pH value studied here (pH 9.0), the surface complex formed at pH 9.0 was most likely a non-protonated bidentate binuclear complex with C_2ν_ symmetry or a monoprotonated monodentate complex (C_1_). From our infrared spectra it was not possible to differentiate between these two types of complexes. At this high pH there was no indication of precipitated AlPO_4_ probably due to an increase in the solubility of aluminium phosphate with increasing pH in combination with the detection limit of the spectroscopic method used.

The adsorption of phosphate at the two pH conditions could be well described with the Langmuir type of adsorption isotherm at low concentrations and the empirical Freundlich adsorption isotherm for the whole concentration range, *viz.* 5 – 2000 μM.

## Competing interests

The authors declare that they have no competing interests.

## Authors’ contributions

TZ carried out most experiments; ZS has formulated the research idea, drafted the manuscript and finalized the manuscript; XY has assisted to carry out the measurement of ATR-FTIR; AH has assisted to write considerable amount of text and explained the ATR-FTIR spectra. All authors read and approved the final manuscript.

## Supplementary Material

Additional file 1 **Figure S1.** Adsorption/desorption isotherms and pore diameter distribution of the synthesized mesoporous alumina sample. Figure S2. XRD results of the synthesized mesoporous alumina sample, which shows clearly the characteristic peaks of gamma-Al2O3 (PDF No. 79-1558). Figure S3. Distribution of aqueous H_n_PO_4_^n-3^ species at different pH, it can be seen that at pH~4, the dominating solution species is H_2_PO_4_^-^ and at pH~9 the dominating solution species is HPO_4_^2-^. Figure S4. Surface species distribution of aluminum oxide in solution. Click here for file
