# Supplementary material for: Sorption of phosphate onto mesoporous γ-alumina studied with in-situ ATR-FTIR spectroscopy
Source: Chem Cent J. 2012 Apr 3;6:26. doi: 10.1186/1752-153X-6-26 (PMC3441211; doi:10.1186/1752-153X-6-26)
Supplement: Additional file 1 — Figure S1. Adsorption/desorption isotherms and pore diameter distribution of the synthesized mesoporous alumina sample. Figure S2. XRD results of the synthesized mesoporous alumina sample, which shows clearly the characteristic peaks of gamma-Al2O3 (PDF No. 79-1558). Figure S3. Distribution of aqueous HnPO4n-3 species at different pH, it can be seen that at pH~4, the dominating solution species is H2PO4- and at pH~9 the dominating solution species is HPO42-. Figure S4. Surface species distribution of aluminum oxide in solution. [file 1752-153X-6-26-S1.docx]

# Sorption of phosphate onto mesoporous *γ*-alumina studied with in-situ ATR-FTIR spectroscopy

Ting-Ting Zheng^†,‡^, Zhong-Xi Sun^*,†^ , Xiao-Fang Yang^§,‡^ and Allan Holmgren^*,‡^

^†^School of Chemistry and Chemical Engineering, University of Jinan, 250022 Jinan, China

^‡^Division of Chemical Engineering, Luleå University of Technology, S- 971 87 Luleå, Sweden

^§^ Research Center for Eco-Environmental Sciences, Chinese Academy of Sciences, Beijing, China

^*^ Corresponding author. Email: [sunzx@ujn.edu.cn](mailto:sunzx@ujn.edu.cn), allan.r.holmgren@ltu.se. Tel: +86 (0)531 82765426, +46 (0)920 492140. Fax: +86 (0)531 7161600, +46 (0)920 491199.

**Supporting Information**

Figure S1. Adsorption/desorption isotherms and pore diameter distribution of the synthesized mesoporous alumina sample

Figure S2. XRD results of the synthesized mesoporous alumina sample, which shows clearly the characteristic peaks of gamma-Al_2_O_3_ (PDF No. 79-1558)

Figure S3. Distribution of aqueous H_n_PO_4_^3-n^ species at different pH, it can be seen that at pH~4, the dominating solution species is H_2_PO_4_^-^ and at pH~9 the dominating solution species is HPO_4_^2-^.

Figure S4. Surface species distribution of aluminum oxide in solution

(Log K_1_ = 3.90 and Log K_2_ = -8.48 obtained using CCM model; parameter used are ionic strength 0.1M NaNO_3_; ≡AlOH = 1000μM; C = 4 F/m^2^)*

*Liu J., Zhang WM, Wu ZS, Qin LH, Sun RG, Sun ZX: **Surface acid base properties and adsorption behaviors of heavy metal ions in aqueous suspension of α-Fe_2_O_3_，γ-Al_2_O_3_ and their mixed systems**，Chinese Journal of Inorganic Chemistry 2010, **26**: 1967-1974.

Figure S5. Figure S5. Infrared spectra of phosphate adsorbed onto γ-alumina from 10, 25, 50, 200, 500, 1000, and 2000 μM aqueous phosphate solutions (from bottom to top). Spectra were not baseline corrected or smoothed. The time of adsorption was 10 minutes at each concentration.

Figure S1

Figure S2

Figure S3

Figure S4

Figure S5
